# Association between Asthma and Oral Health Symptoms in Adolescents

**DOI:** 10.3390/ijerph20042921

**Published:** 2023-02-07

**Authors:** Ka-Yun Sim, Yun-Seo Jang, Na-Young Yoon, Eun-Cheol Park

**Affiliations:** 1 Department of Public Health, Graduate School, Yonsei University, Seoul 03722, Republic of Korea; 2 Institute of Health Services Research, Yonsei University, Seoul 03722, Republic of Korea; 3 Department of Preventive Medicine, Yonsei University College of Medicine, Seoul 03722, Republic of Korea

**Keywords:** oral health symptoms, asthma, adolescents, health behavior

## Abstract

Oral health is an indicator of patients’ overall quality of life. Poor oral health among adolescents with asthma can affect their health in adulthood. This study researched the association between asthma and oral health symptoms in South Korean adolescents. Data from the 2020 Korea Youth Risk Behavior Web-based Survey were used. A total of 44,940 students participated in this study. The dependent variables were self-reported oral health symptoms. Asthma was the primary independent variable based on diagnosis in the past 12 months. The chi-squared test and multivariable logistic regression analysis were used. Students with asthma were associated with oral health symptoms, compared with those without asthma (boys, odds ratio (OR): 1.29, 95% confidence interval (CI) = 1.01–1.66; girls, OR: 1.94, 95% CI = 1.40–2.69). Poor health habits, such as low physical activity, higher sweetened beverage consumption, and fewer sleeping hours, were associated with oral health symptoms. Students who did not receive asthma treatment also had higher oral health symptoms (boys, OR: 1.29, 95% CI = 1.13–1.48, girls, OR: 1.34, 95% CI = 1.15–1.57). Students with absence due to asthma had a higher risk of oral health than those without asthma (boys, OR: 1.31, 95% CI = 1.17–1.46, girls OR: 1.28, 95% CI = 1.12–1.46). Students with asthma had a high risk of poor oral health among South Korean adolescents, suggesting more attention be given to regular dental check-ups and maintaining oral hygiene.

## 1. Introduction

Oral health is an important marker for overall health and quality of life [1]. Since oral health includes a range of diseases and conditions, from dental caries to oral cancer, it has an extensive impact on various aspects of life [2]. According to the 2019 Global Burden of Disease study, oral diseases affect nearly 3.5 billion individuals worldwide [3]. Poor oral health is not only associated with problems such as nutritional abnormalities [4], but is also related to severe health outcomes, such as respiratory diseases, cardiovascular diseases, and infections [5,6,7].

Asthma is the most common chronic disease in children [8]. It is well known as a major noncommunicable disease and affects the long-term health of both children and adults [9]. The World Health Organization reported that asthma caused 455,000 deaths and affected approximately 262 million people in 2019 [10].

The association between asthma and many general diseases, including major causes of death, has been extensively reported. A study in Taiwan reported that participants with bronchial asthma may have an increased risk of developing chronic kidney disease [11]. According to another study, children with asthma are more likely to have higher levels of blood insulin and triglycerides than those without asthma [12]. A Korean study reported an association between asthma and depression in adults [13].

Many previous studies have mainly used children as subjects. Children’s oral health is affected by their parents [14,15,16]. A Japanese study reported that parents’ oral health behavior could affect their children’s condition, including dental health and tooth decay [17]. Some studies have reported a link between asthma and oral health, focusing on patients taking drugs such as inhalers. Children using inhalers may have a higher risk of oral disease than those without asthma [18,19]. These studies included only small sample sizes and were complex by analyzing the effects of childhood asthma on various symptoms of oral health rather than specifically focusing on oral health and asthma. However, some studies used large-scale samples. A study in Korea reported that three diseases, such as asthma, allergic rhinitis, and atopic dermatitis, were related to oral disease [20]. Another study found a link between tooth loss according to age group in asthmatic patients [21].

Adolescence is a period of significant physical, psychological, and behavioral changes that lead to the development of healthy behavior that can last until adulthood [22]. Adolescents have unique risk factors associated with poor oral health and nutritional habits, such as junk food intake and nicotine experiments [23]. More research is needed to investigate the relationship between asthma, which affects overall quality of life, and oral health, a general health indicator.

Therefore, this study attempted to evaluate the relationship between asthma and oral health symptoms of only adolescents using a national cross-sectional survey with a relatively large sample size. After that, based on the questionnaire, four oral health symptoms were stratified, and sub-groups were further analyzed.

## 2. Methods

### 2.1. Data

Data for this cross-sectional study were obtained from the 2020 Korea Youth Risk Behavior Web-based Survey (KYRBWS). The KYRBWS has been conducted by the Korea Disease Control and Prevention Agency (KDCA) since 2005 to assess the status of health behavior of South Korean adolescents and to identify comparable health indicators internationally. The KDCA Research Ethics Review Board approved the data collection protocols for the KYRBWS. Data are accessible for download from the KDCA website (https://www.kdca.go.kr/yhs/, accessed on 1 January 2023). Thus, this study did not require additional approval from the ethics review board. KYRBWS is a self-reported annual survey that uses a stratified, two-stage, clustered sampling design. The subjects of the survey were adolescents aged 12–18 years from approximately 400 schools nationwide. The adolescents voluntarily and anonymously completed the survey form. The number of participants from the 2020 KYRBWS was 54,948 and data were collected from August to November 2020.

### 2.2. Study Population

The total number of participants surveyed was 54,948 in 2020. A total of 8473 students who had missing values were excluded (4196 boys and 4277 girls). Additionally, we excluded 1485 students who responded that they had oral health symptoms due to exercise or accidents (999 boys and 486 girls). As a result, we excluded 9958 students (5195 boys and 4763 girls). A total of 457 students had asthma in the study population. Among them, 266 responded that they had oral health symptoms in the past 12 months. Finally, 44,990 samples (23,461 without symptoms and 21,529 with symptoms) were analyzed.

### 2.3. Variables

The dependent variable was self-reported oral health symptoms. The KYRBWS assesses oral health symptoms based on the question, “Have you experienced the following symptoms in the last 12 months?” The survey inquired about the following symptoms: (1) chipped or broken tooth, (2) toothache when eating or drinking, (3) throbbing and sore teeth, and (4) sore and bleeding gums. We divided the participants into two groups according to those who answered “yes” to more than one of the questions (defined as the “oral health symptom group”) and those who answered “no” to all questions (defined as the “oral health symptomless group”).

Asthma was the main independent variable in this study. Participants were asked about their history of asthma based on the question, “Have you ever been diagnosed with asthma by a doctor in the past 12 months?” Study participants were classified into either the asthma group if they answered “yes” or the non-asthma group if they answered “no”.

Covariates that could act as potential confounding factors were controlled. These covariates included socioeconomic factors and behavioral health patterns. Students were divided into six groups, from middle school to high school. Household income, region, and perceived stress level were divided into three groups. Smoking and alcohol status was divided into two groups, depending on whether they experienced it. Physical activity levels were divided into high and low groups. Adolescents should do more than 60 min of intense physical activity daily, including aerobic exercise, for at least three days a week [24,25]. Therefore, students with vigorous aerobic activity for at least three days were classified as a group with high physical activity. Students were asked about drinking soda or sweet drinks: Have they had soda or sweet drinks in the past seven days? In the case of sleep duration, we divided the participants into two groups based on an eight-hour duration, of which is recommended for adolescents by the National Sleep Foundation in the United States of America [26]. Teeth brushing frequency was based on the previous day. We used treatment status and school absence as variables to investigate the severity of asthma. We divided treatment status into three groups based on the last 12 months. School absences were divided into groups of less than three days and more than four days.

### 2.4. Statistical Analysis

A chi-squared test was conducted to investigate the general characteristics of the study population. The general characteristics of the sample were represented by frequency and percentage and were based on descriptive analysis. To assess the relationship between asthma and oral health symptoms in adolescents, we used multivariable logistic regression analysis with covariate adjustment. Subgroup analyses were conducted to determine the detailed effects of oral health symptoms on asthma and were stratified by sex. All results are presented as odds ratios (OR) and 95% confidence intervals (CI). The analyses were performed using stratified sampling variables. All estimates were generated using weighted variables to generalize the data. All statistical analyses were conducted using the SAS version 9.4 software (SAS Institute, Cary, NC, USA). Statistical significance was determined as a two-sided *p*-value < 0.05.

## 3. Results

### 3.1. General Characteristics

Table 1 summarizes the characteristics of the study population. Of the 44,940 participants, 23,461 (52.1%) had no oral health symptoms and 21,529 (47.9%) had oral health symptoms. Among 457 with asthma, 266 (1.2%) answered that they had oral health symptoms.

### 3.2. Logistic Regression Results

Table 2 shows the multivariate logistic regression analysis results on the association between asthma and oral health symptoms after adjustment for all covariates. Asthma groups were significantly associated with oral health symptoms regardless of gender (boys OR: 1.29, 95% CI = 1.01–1.66, girls OR: 1.94, 95% CI = 1.40–2.69). The risk of oral health symptoms was high in students who consumed sweetened beverages during the past week (boys OR: 1.16, 95% CI = 1.06–1.26, girls OR: 1.25, 95% CI = 1.16–1.36). Students who frequently exercised and followed the recommended sleep time were at low risk of developing oral health symptoms regardless of gender.

### 3.3. Results of an Independent Variable Sub-Analysis 

Table 3 shows the analysis results of independent variables representing the OR of oral health symptoms stratified by asthma. Among students with asthma, those who did not follow the recommendation for physical activity had a risk of oral health symptoms (boys OR: 1.33, 95% CI = 1.02–1.72, girls OR: 2.03, 95% CI = 1.45–2.86). The risk of oral health symptoms was higher in asthmatic students who drank more sweetened beverages (boys OR: 1.44, 95% CI = 1.09–1.90, girls OR: 1.99, 95% CI = 1.37–2.88).

### 3.4. Results of Dependent Variable Sub-Analysis 

**Figure 1:**
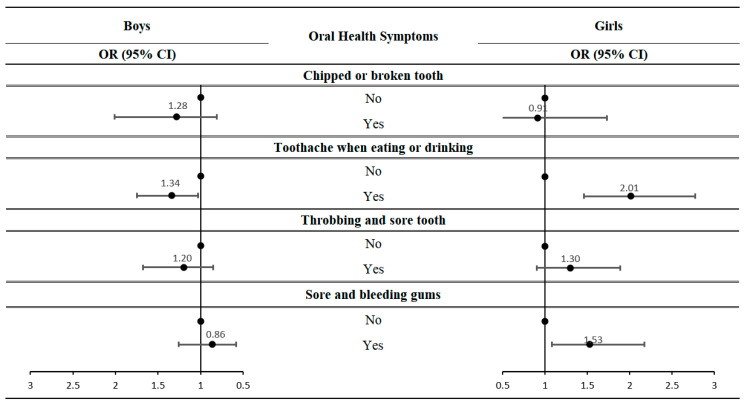
Association of oral health symptoms with asthma, with non-asthma students as the reference category.

Figure 1 presents the findings of the subgroup analysis, stratified by the dependent variable, using a questionnaire on oral health symptoms. Students with asthma were more likely to experience toothache when eating or drinking than those without asthma (boys OR: 1.34, 95% CI = 1.03–1.75, girls OR: 2.01, 95% CI = 1.46–2.77). In female students, asthma students had a higher risk of soreness and bleeding gum than those without asthma (OR: 1.53, 95% CI = 1.08–2.17).

### 3.5. The Severity of Asthma

Table 4 presents the severity of asthma through treatment status and school absence. Male students who received irregular treatment had a high risk of oral health symptoms (OR: 1.43, 95% CI = 1.18–1.74), and students who did not receive treatment, regardless of gender, had a high risk of oral health symptoms (boys OR: 1.29, 95% CI = 1.13–1.48, girls OR: 1.34, 95% CI = 1.15–1.57). Students who experienced absence for 1 to 3 days due to asthma had a higher risk of oral health symptoms than those without asthma (boys OR: 1.31, 95% CI = 1.17–1.46, girls OR: 1.28, 95% CI = 1.12–1.46). In the case of prolonged absences over four days, it was not statistically significant.

## 4. Discussion

Based on self-reported data, this study aimed to identify whether asthma is associated with oral health symptoms among South Korean adolescents. The overall findings of this study showed that adolescents with asthma were associated with oral health symptoms, regardless of sex. In the subgroup analysis stratified as a dependent variable, male students were statistically significant only in toothache symptoms, and female students showed a significant association with toothache and bleeding gums. In addition, the risk of oral health was higher in students who were treated irregularly or not treated at all, and the risk was higher in students who experienced absences from asthma.

The mechanisms underlying the relationship between asthma and oral health have been described in previous studies. Poor oral health is a potential risk factor for systemic inflammatory diseases, including allergic diseases such as asthma. The association between allergic diseases and poor oral health is related to increased levels of inflammatory cytokines, such as interleukin (IL)-1β, IL-6, and tumor necrosis factor-α. Although the physiological mechanism underlying the association between allergic diseases and poor oral health is not clear, inflammation of periodontal tissues or oral pathogens is thought to activate host immune cells, releasing inflammatory cytokines [20,27].

According to a review article published in 2022, children with asthma tend to have mouth-breathing habits, owing to frequent airway obstruction. This can result in dehydration of the oral mucosa [22]. Moreover, asthma medications, such as beta-2 agonists, can reduce salivary flow rates, which can inhibit the ability of the saliva to clear the oral cavity and diminish its buffering capacity [28].

As shown in Table 2, students with high levels of physical activity and relatively long sleep time had a low risk of oral health symptoms. Students who responded that they drank sweetened beverages were more likely to experience a higher risk of developing oral health symptoms. In Table 3, the risk of oral health symptoms was also higher in students who were less physically active and drank more sweetened beverages. These results can be explained by their way of life, where students with asthma often have restrictions on school life and sports; hence, families tend to be generous in allowing them to eat sweets [22]. They also develop mouth-breathing, and thirst is caused by dehydration [28]. They may consume sweetened beverages regularly to compensate for a dry mouth and to wash away the bitter medication taste [22]. Frequent consumption of sweetened drinks due to extreme thirst can also be a reason for the increase in oral diseases, such as dental caries, in patients with asthma [28]. In addition, some studies have reported that adolescents with severe asthma experience significant insomnia and insufficient sleep [29,30]. Overall, we suggested that improving health habits can help manage the risk of oral health symptoms in patients with asthma.

Students with asthma were more likely to experience toothache when eating or drinking, regardless of gender. In addition, the OR value of female students tended to be higher than male students. According to some previous studies, dental caries is the most significant cause of toothache [31,32]. Females generally tend to develop more dental caries relative to males [33]. The reasons include increased exposure to the infectious oral environment due to an earlier eruption of teeth in females, hormonal effects, and genetic predispositions, such as variations in the X-linked amelogenin gene [34]. Hormone levels related to menstruation and pregnancy affect the periodontium and the biochemical composition and overall flow rate of saliva, resulting in a more cariogenic oral environment for females than for males. Approximately 90% of amelogenin in the body is expressed by the X-chromosome; deficiencies in amelogenin can disrupt the hard enamel tooth surface, thus increasing the susceptibility to dental caries [34]. This study reported that the results tended to be significant in female students, which can be explained by the epidemiology of asthma. As the size of the lungs of males in their childhood is smaller than that of the airway, the prevalence of childhood asthma is higher in males [22]. However, this phenomenon is reversed in adolescence [22]. In terms of reversed situations during adolescence, epidemiologically, it is likely that the results for female students with asthma were more sensitively affected indirectly.

Previous studies on periodontal disease explain sore and bleeding gum risk in asthma patients. One study suggested that gingivitis in patients with asthma could be caused by an altered immune response and dehydration related to mouth-breathing. Another study reported that the concentration of immunoglobulin E in gum tissue may increase, causing periodontal destruction, such as signs of sore and bleeding gums [22].

In Table 4, among male students with asthma, those who irregularly received or did not receive at all treatment had a higher risk of oral health symptoms than those without asthma. Female students with untreated asthma also had higher oral health symptoms. Both male and female students showed no significant long-term absence for more than four days, and students absent for less than three days showed a risk of oral health compared to students without asthma, regardless of gender. Additionally, our results showed that students with absence due to asthma had a higher risk of oral health than students without asthma.

According to previous studies, asthma remission generally refers to a condition, which is an absence of respiratory symptoms without using anti-asthma medications for more than two years [35,36]. Irregular treatment affects lung functions, increasing the risk of developing chronic obstructive pulmonary disease (COPD) [37,38]. Asthma may diminish ventilation functions of the lower respiratory tract, inducing mucosal congestion, edema, and mucus plugs formation [39]. If treated irregularly or completely untreated, asthma worsens, leading to decreased salivary flow and increased thirst, threatening oral health. Asthma is the most common cause of school absences [40], and frequent absences can significantly impact school performance [41]. In particular, vulnerable groups such as low-income families can lead to a cycle of lifelong academic achievement [41]. Preemptive asthma management and regular treatment are essential to prevent this vicious cycle.

This study had several limitations. Firstly, it was a cross-sectional study. It is difficult to identify an inverse causal relationship; therefore, caution should be exercised in the interpretation of results. Secondly, the values may not be accurately measured since the KYRBWS data were collected through a self-reported survey. Our criteria for asthma diagnosis were conducted by reviewing previous studies, but subsequent studies should consider examination data for the accurate diagnosis of asthma. In addition, using indicators that quantitatively measure oral health, such as DMFT indicators (decayed, filled, and missing teeth) [42], should be considered. Third, it was relatively simple to use treatment status and school absences to measure the severity of asthma. Further studies require the consideration of medical records.

Despite these limitations, our study had several strengths. First, the study was based on the KYRBWS, a nationally representative dataset collected by the KDCA. This is helpful for health-related research since it is conducted annually to reflect changes in the health conditions of Korean adolescents. It is performed using a representative random cluster design, which can generalize the findings of the study to a general population. Second, although the KYRBWS contains self-reported data since it is an anonymous web-based survey, it is likely to acquire relatively trustworthy responses. Third, it has the advantage of identifying the risk of oral health symptoms exclusively for adolescents, not including adults.

## 5. Conclusions

The association between asthma and oral health symptoms among South Korean adolescents was demonstrated in this study. We emphasize that students with asthma should pay more attention to regular dental checkups and maintain oral hygiene. Adolescents with asthma must take care of oral health while receiving regular asthma treatment. This study can be considered a baseline for developing useful policies to improve oral health among adolescents. Further prospective studies are required to identify the association between asthma and oral health symptoms.

## Figures and Tables

**Table 1:** General characteristics of the study population.

Variables		Oral Health Symptoms						
		Total		No		Yes		*p*-Value
		*N*	%	*N*	%	*N*	%	
Total		44,990	100.0	23,461	52.1	21,529	47.9	
Diagnosis of Asthma								<0.0001 *
	No	44,533	99.0	23,270	99.2	21,263	98.8	
	Yes	457	1.0	191	0.8	266	1.2	
Sex								<0.0001 *
	Boys	23,158	51.5	13,213	56.3	9945	46.2	
	Girls	21,832	48.5	10,248	43.7	11,584	53.8	
Age (Grade)								<0.0001 *
	7th	8377	18.6	4920	21.0	3457	16.1	
	8th	7684	17.1	4158	17.7	3526	16.4	
	9th	7360	16.4	3772	16.1	3588	16.7	
	10th	7280	16.2	3753	16.0	3527	16.4	
	11th	7268	16.2	3570	15.2	3698	17.2	
	12th	7021	15.6	3288	14.0	3733	17.3	
Household Income								<0.0001 *
	Low	5775	12.8	2556	10.9	3219	15.0	
	Middle	21,655	48.1	11,252	48.0	10,403	48.3	
	High	17,560	39.0	9653	41.1	7907	36.7	
Region								0.0341 *
	Metropolitan	19,320	42.9	10,211	43.5	9109	42.3	
	Urban	22,034	49.0	11,370	48.5	10,664	49.5	
	Rural	3636	8.1	1880	8.0	1756	8.2	
Smoking Status								<0.0001 *
	Never	40,843	90.8	21,661	92.3	19,182	89.1	
	Ever	4147	9.2	1800	7.7	2347	10.9	
Alcohol Status								<0.0001 *
	Never	30,456	67.7	16,859	71.9	13,597	63.2	
	Ever	14,534	32.3	6602	28.1	7932	36.8	
Physical Activity								<0.0001 *
	Low	42,975	95.5	22,224	94.7	20,751	96.4	
	High	2015	4.5	1237	5.3	778	3.6	
Perceived Stress Level								<0.0001 *
	Low	9841	21.9	6304	26.9	3537	16.4	
	Middle	20,198	44.9	10,674	45.5	9524	44.2	
	High	14,951	33.2	6483	27.6	8468	39.3	
Drinking Soda								<0.0001 *
	No	10,211	22.7	5543	23.6	4668	21.7	
	Yes	34,779	77.3	17,918	76.4	16,861	78.3	
Drinking Sweetened Beverage								<0.0001 *
	No	7629	17.0	4341	18.5	3288	15.3	
	Yes	37,361	83.0	19,120	81.5	18,241	84.7	
Sleep Duration								<0.0001 *
	Adequate	34,465	76.6	17,192	73.3	17,273	80.2	
	Inadequate	10,525	23.4	6269	26.7	4256	19.8	
Teeth Brushing Frequency								<0.0001 *
	0 times a day	347	0.8	140	0.6	207	1.0	
	1 time	3041	6.8	1337	5.7	1704	7.9	
	2 times	19,701	43.8	10,067	42.9	9634	44.7	
	3 times	17,164	38.2	9387	40.0	7777	36.1	
	4 times	4737	10.5	2530	10.8	2207	10.3	

* *p*-value < 0.05.

**Table 2:** Results of factors associated between asthma and oral health symptoms.

Variables		Oral Health Symptoms			
		Boys		Girls	
		OR	95% CI	OR	95% CI
Diagnosis of Asthma					
	No	1.00		1.00	
	Yes	1.29 *	(1.01–1.66)	1.94 *	(1.40–2.69)
Age (Grade)					
	7th (middle school)	1.00		1.00	
	8th	1.03	(0.94–1.14)	1.20 *	(1.08–1.33)
	9th	1.15 *	(1.04–1.27)	1.34 *	(1.21–1.50)
	10th (high school)	1.03	(0.92–1.24)	1.21 *	(1.09–1.34)
	11th	1.01	(0.90–1.13)	1.32 *	(1.18–1.48)
	12th	1.10	(0.98–1.24)	1.35 *	(1.20–1.52)
Household Income					
	Low	1.00		1.00	
	Middle	0.81 *	(0.73–0.89)	0.83 *	(0.75–0.91)
	High	0.82 *	(0.74–0.90)	0.79 *	(0.72–0.86)
Region					
	Metropolitan	1.00		1.00	
	Urban	1.07 *	(1.00–1.14)	0.99	(0.93–1.06)
	Rural	0.97	(0.86–1.10)	0.96	(0.84–1.11)
Smoking Status					
	Never	1.00		1.00	
	Ever	1.20 *	(1.09–1.31)	1.22 *	(1.06–1.40)
Alcohol Status					
	Never	1.00		1.00	
	Ever	1.25 *	(1.17–1.33)	1.40 *	(1.29–1.51)
Physical Activity					
	Low	1.00		1.00	
	High	0.76 *	(0.68–0.85)	0.84	(0.66–1.07)
Perceived Stress Level					
	Low	1.00		1.00	
	Middle	1.43 *	(1.33–1.54)	1.45 *	(1.33–1.58)
	High	1.85 *	(1.71–2.01)	2.00 *	(1.83–2.18)
Drinking Soda					
	No	1.00		1.00	
	Yes	1.04	(0.96–1.12)	1.11 *	(1.04–1.19)
Drinking Sweetened Beverage					
	No	1.00		1.00	
	Yes	1.16 *	(1.06–1.26)	1.25 *	(1.16–1.36)
Sleep Duration					
	Adequate	1.00		1.00	
	Inadequate	0.83 *	(0.78–0.89)	0.80 *	(0.74–0.87)
Teeth Brushing Frequency					
	0 times a day	1.00		1.00	
	1 time	0.89	(0.68–1.17)	0.95	(0.57–1.59)
	2 times	0.68 *	(0.53–0.90)	0.69	(0.42–1.13)
	3 times	0.55 *	(0.43–0.72)	0.58 *	(0.36–0.96)
	4 times	0.57 *	(0.43–0.76)	0.64	(0.38–1.05)

* *p*-value < 0.05.

**Table 3:** Results of subgroup analysis stratified by independent variables.

Variables		Oral Health Symptoms					
		Boys			Girls		
		Non	Asthma		Non	Asthma	
		OR	OR	95% CI	OR	OR	95% CI
Age (Grade)							
	7th (middle school)	1.00	1.30	(0.68–2.48)	1.00	1.95	(0.87–4.35)
	8th	1.00	1.75	(0.94–3.23)	1.00	2.05	(0.98–4.27)
	9th	1.00	0.80	(0.41–1.56)	1.00	0.76	(0.35–1.66)
	10th (high school)	1.00	1.58	(0.61–4.07)	1.00	2.96 *	(1.14–7.68)
	11th	1.00	1.56	(0.79–3.09)	1.00	3.06 *	(1.15–8.16)
	12th	1.00	1.04	(0.61–1.77)	1.00	2.19	(0.87–5.54)
Household Income							
	Low	1.00	1.07	(0.52–2.20)	1.00	2.08	(0.78–5.62)
	Middle	1.00	1.47	(0.97–2.24)	1.00	2.18 *	(1.41–3.37)
	High	1.00	1.23	(0.83–1.82)	1.00	1.57	(0.86–2.86)
Region							
	Metropolitan	1.00	0.81	(0.57–1.14)	1.00	2.41	(1.44–4.03)
	Urban	1.00	1.88 *	(1.30–2.71)	1.00	1.69 *	(1.08–2.66)
	Rural	1.00	1.18	(0.44–3.21)	1.00	1.54	(0.24–9.86)
Smoking Status							
	Never	1.00	1.29	(0.98–1.69)	1.00	1.83 *	(1.31–2.57)
	Ever	1.00	1.33	(0.67–2.66)	1.00	-	-
Alcohol Status							
	Never	1.00	1.36	(0.97–1.90)	1.00	1.73 *	(1.18–2.54)
	Ever	1.00	1.20	(0.79–1.80)	1.00	3.30 *	(1.60–6.79)
Physical Activity							
	Low	1.00	1.33 *	(1.02–1.72)	1.00	2.03 *	(1.45–2.86)
	High	1.00	0.81	(0.30–2.25)	1.00	0.54	(0.07–4.29)
Perceived Stress level							
	Low	1.00	1.03	(0.56–1.89)	1.00	2.22	(0.77–6.46)
	Middle	1.00	1.61 *	(1.06–2.44)	1.00	2.20 *	(1.34–3.60)
	High	1.00	1.14	(0.75–1.75)	1.00	1.68 *	(1.04–2.72)
Drinking Soda							
	No	1.00	1.36	(0.73–2.55)	1.00	1.96 *	(1.07–3.58)
	Yes	1.00	1.28	(0.97–1.69)	1.00	1.95 *	(1.32–2.87)
Drinking Sweetened beverage							
	No	1.00	0.73	(0.32–1.64)	1.00	1.82	(0.83–4.00)
	Yes	1.00	1.44 *	(1.09–1.90)	1.00	1.99 *	(1.37–2.88)
Sleep Duration							
	Adequate	1.00	1.30	(0.98–1.71)	1.00	1.88 *	(1.29–2.74)
	Inadequate	1.00	1.25	(0.67–2.31)	1.00	2.26 *	(1.03–4.92)
Teeth Brushing Frequency							
	0 times a day	1.00	1.45	(0.11–18.40)	1.00	0.46	(0.10–2.03)
	1 time	1.00	1.24	(0.46–3.39)	1.00	1.30	(0.30–5.62)
	2 times	1.00	1.53 *	(1.06–2.22)	1.00	1.52	(0.93–2.49)
	3 times	1.00	1.02	(0.65–1.59)	1.00	2.54 *	(1.54–4.19)
	4 times	1.00	1.45	(0.59–3.58)	1.00	1.74	(0.64–4.74)

* *p*-value < 0.05.

**Table 4:** Results of subgroup analysis stratified by the severity of asthma.

Variables		Oral Health Symptoms			
		Boys		Girls	
		OR	95% CI	OR	95% CI
Treatment Status					
	No asthma	1.00		1.00	
	Regularly	1.00	(0.66–1.53)	1.30	(0.73–2.30)
	Irregularly	1.43 *	(1.18–1.74)	1.16	(0.92–1.47)
	Never	1.29 *	(1.13–1.48)	1.34 *	(1.15–1.57)
School Absence					
	No asthma	1.00		1.00	
	≤3 days	1.31 *	(1.17–1.46)	1.28 *	(1.12–1.46)
	≥4 days	1.38	(0.62–3.05)	1.24	(0.55–2.80)

* *p*-value < 0.05.

## Data Availability

The dataset used in this study is publicly accessible (https://www.kdca.go.kr/yhs/ (accessed on 1 January 2023)).
